# Malaria during pregnancy and foetal haematological status in Blantyre, Malawi

**DOI:** 10.1186/1475-2875-4-39

**Published:** 2005-08-25

**Authors:** Elizabeth T Abrams, Jesse J Kwiek, Victor Mwapasa, Deborah D Kamwendo, Eyob Tadesse, Valentino M Lema, Malcolm E Molyneux, Stephen J Rogerson, Steven R Meshnick

**Affiliations:** 1 Department of Humanities and Social Sciences, California Institute of Technology, Pasadena, California, USA; 2 Department of Epidemiology, University of North Carolina, Chapel Hill, North Carolina, USA; 3 Department of Community Health, University College of Medicine, University of Malawi, Blantyre, Malawi; 4 UNC Project, Lilongwe, Malawi; 5 Department of Obstetrics and Gynaecology, College of Medicine, University of Malawi, Blantyre, Malawi; 6 School of Tropical Medicine, University of Liverpool, Liverpool, UK; 7 Malawi-Liverpool-Wellcome Trust Clinical Research Programme, College of Medicine, University of Malawi, Blantyre, Malawi; 8 Department of Medicine, University of Melbourne, Royal Melbourne Hospital, Parkville, Australia

## Abstract

**Background:**

Although maternal anaemia often stems from malaria infection during pregnancy, its effects on foetal haemoglobin levels are not straightforward. Lower-than-expected cord haemoglobin values in malarious versus non-malarious regions were noted by one review, which hypothesized they resulted from foetal immune activation to maternal malaria. This study addressed this idea by examining cord haemoglobin levels in relation to maternal malaria, anaemia, and markers of foetal immune activation.

**Methods:**

Cord haemoglobin levels were examined in 32 malaria-infected and 58 uninfected women in Blantyre, Malawi, in relation to maternal haemoglobin levels, malaria status, and markers of foetal haematological status, hypoxia, and inflammation, including TNF-α, TGF-β, and ferritin. All women were HIV-negative.

**Results:**

Although malaria was associated with a reduction in maternal haemoglobin (10.8 g/dL vs. 12.1 g/dL, p < 0.001), no reduction in cord haemoglobin and no significant relationship between maternal and cord haemoglobin levels were found. Cord blood markers of haematological and hypoxic statuses did not differ between malaria-infected and uninfected women. Maternal malaria was associated with decreased TGF-β and increased cord ferritin, the latter of which was positively correlated with parasitaemia (r = 0.474, p = 0.009). Increased cord ferritin was associated with significantly decreased birth weight and gestational length, although maternal and cord haemoglobin levels and malaria status had no effect on birth outcome.

**Conclusion:**

In this population, cord haemoglobin levels were protected from the effect of maternal malaria. However, decreased TGF-β and elevated ferritin levels in cord blood suggest foetal immune activation to maternal malaria, which may help explain poor birth outcomes.

## Background

Malaria affects more than three million pregnant women per year in developing countries, where it commonly causes poor birth outcomes and maternal anaemia [1-3]. Brabin [4] suggested that foetal anaemia also resulted from maternal malaria during pregnancy. In developed countries, anaemia in newborns is rare, regardless of maternal status, and normal neonatal haemoglobin levels in these populations are higher than adult levels [e.g. [5]] In addition, there appears to be little relationship between maternal and umbilical cord haemoglobin levels, even when mothers are anaemic [e.g., [5,6]]. For example, a study of pregnant Turkish women found no significant difference in mean cord haemoglobin levels of neonates of anaemic (Hb < 10 g/dL) mothers (mean ± SEM: 16.11 ± 0.39 g/dL) compared to those of non-anaemic (Hb ≥ 12 g/dL) mothers (mean ± SEM: 16.57 ± 1.35 g/dL) [5]. In developing countries, the situation is more complex. In non-malarious areas, babies of mothers with iron-deficient anaemia have both relatively similar [e.g., [7]] and higher [e.g., [8,9]] cord haemoglobin levels than those reported for developed countries. In contrast, cord haemoglobin levels in malarious areas have been characterized by Brabin [4] as lower-than-expected, hypothesized to result from foetal immune activation to maternal malarial antigens.

In non-malarious regions, maternal anaemia may stem chiefly from iron deficiency; higher cord haemoglobin levels may thus reflect a response to foetal hypoxia caused by decreased placental oxygen transport [10]. In malarious regions, however, malaria infections during pregnancy present a second source of maternal anaemia by increasing red blood cell destruction and decreasing erythropoiesis [3,11,12]. These effects may be mediated in part by elevations in proinflammatory cytokines like TNF-α, which are associated with maternal anaemia in malaria-infected pregnancies [13,14]. Although malaria elevates placental proinflammatory cytokine production during pregnancy [14-16], it has not been reported to affect cord levels [15]. However, maternal malaria may immunologically prime the foetus, as several researchers have reported that foetal explants produce proinflammatory cytokines upon exposure to maternal malarial antigens [17,18].

To further address the hypothesis that cord haemoglobin levels in malarious areas are lower than expected due to foetal immune activation to maternal malaria, cord haemoglobin levels were examined in this study in malaria-infected and uninfected women in relation to maternal malaria status and haemoglobin levels and markers of foetal haematological, inflammatory, and hypoxic status. In specific, chronic foetal hypoxia was assessed via levels of erythropoietin (Epo) [19], a hormone responsible for red blood cell production, and corticotrophin releasing hormone (CRH) and cortisol, two hormones that have been hypothesized to mediate the effects of anaemia-related hypoxia on poor birth outcomes [20]. Tumor necrosis factor alpha (TNF-α), a pro-inflammatory cytokine, transforming growth factor beta (TGF-β), an anti-inflammatory cytokine, and C-reactive protein (CRP), an acute shock protein, were assayed as markers of inflammatory status. Haematological status was assessed via umbilical cord levels of haemoglobin (Hb) and soluble transferrin receptor (sTfR). Levels of ferritin, an iron storage protein that is an acute phase reactant in the presence of infection, were also assayed.

As markers of interest were measured both in maternal peripheral and umbilical cord blood, one critical issue is whether the cord measures truly reflect foetal production rather than placental transfer of maternally produced factors. Human data addressing this topic is limited, given the invasive and/or complex nature of the required studies. In general, studies suggest that most substances can cross the placenta, with the rate dependent on molecular weight, such that those with larger molecular weights cross slowly and those with molecular weights under 500 daltons quite rapidly [21]. Erythropoietin, a protein with a large molecular weight, is undetectable on the placental side opposite its origin [22], and it is likely that high molecular weight sequesters other large molecules like ferritin [23] on the side of the placenta on which they were produced. A placental perfusion study of inflammatory cytokine transfer suggested that TNF-α, a cytokine measured in this study, does not readily cross from the maternal to the foetal side of the placenta, although IL-6, which was not assayed in this study, appears to cross the placenta bidirectionally [24]. CRP is also unlikely to cross the placenta [25]. Cortisol may cross the placenta in its active form, but foetal exposure to maternal cortisol is normally limited by the conversion of cortisol to cortisone, its inactive metabolite, by placental 11β-hydroxysteroid dehydrogenase [26].

## Methods

### Subjects

Ninety pregnant women pre-labor or in the latent phase of labor attending the Labour Ward, Queen Elizabeth Central Hospital, Blantyre, Malawi, were recruited from February to October 2002 as part of a prospective cohort study investigating the impact of maternal malaria on HIV vertical transmission [27]. All women with positive peripheral malaria blood smears in the larger study were enrolled, along with the next two sequential uninfected women. Women were excluded from this study if they had HIV, preeclampsia, or multiple gestations.

Maternal socio-economic (marital status, educational level, maternal and paternal occupations, and house construction), health (antenatal clinic attendance and iron tablets usage) and anthropometric (height, weight, and mid-upper arm circumference (MUAC)) data were collected. Neonatal anthropometrics (head, abdominal, and arm circumferences, recumbent length, and weight) were evaluated within the first 24 hours after birth. Gestational age was determined by the New Ballard Score [28].

### Sampling procedure

Maternal peripheral blood samples were taken at recruitment, and cord and placental samples were collected at delivery. Thick blood films were prepared from these samples for malaria microscopy. Placental blood was collected into EDTA by incising the cleaned maternal surface of the placenta and aspirating blood welling from the incision with a sterile pipette. Samples were separated within one hour, and plasma was stored at -70°C. Placental biopsies from a pericentric area (approximately 1 cm from the cord on the maternal side) were placed into 10% neutral buffered formalin to be used for placental malaria histopathology.

### Malaria status

Thick blood films were air-dried and Field's stained. *Plasmodium falciparum *malaria parasitaemia per μl was determined by counting parasites per 200 leukocytes, assuming 6000 leukocytes per μl [29]. Placental malaria histopathology was determined by SJR as described [13]; stage of infection and degree of malaria pigment in placental monocytes and fibrin were evaluated to determine disease severity [13]. For the purposes of data analysis, malaria infection was defined as either maternal (any parasites on maternal peripheral thick blood film) or placental (any parasites on placental thick blood film or histopathology).

### HIV status

All subjects were consented and received HIV pre-test and post-test counseling before the onset of active labor. HIV status was determined by Serocard rapid test for HIV-1 and 2 (Trinity Biotech, Wicklow, Ireland) and Determine HIV1/2 (Abbott Diagnostics, Abbott Park, Illinois, USA); disagreement between tests was settled by HIV-SPOT ELISA (Genelabs Diagnostics, Singapore).

### Markers of haematological status

Haemoglobin (Hb) concentration were measured in peripheral and cord blood samples using a Hemocue^® ^haemoglobinometer (HemoCue AB, Ängelholm, Sweden); some results were not available as samples clotted. Maternal peripheral and cord plasma soluble transferrin receptor (sTfR) and ferritin were assayed by enzyme-linked immunosorbent assay (ELISA) kits according to manufacturer's instructions (Ramco Laboratories, Stafford, TX; and IBL, Hamburg, Germany; respectively). Data were analysed in Microsoft Excel using a log-log standard curve. Limits of detection were as follows: 1 μg/ml (sTfR) and 0.59 ng/ml (ferritin).

### Markers of inflammation

Maternal peripheral, placental and cord plasma tumor necrosis factor-α (TNF-α) and transforming growth factor-β (TGF-β) and maternal peripheral and cord plasma C reactive protein (CRP) levels were also assayed by ELISA (R&D Systems, Minneapolis, MN, USA). Color was developed by SigmaFast o-phenylenediamine dihydrochloride tablets (Sigma, St. Louis, MO, USA). Data were analysed as described above. Limits of detection were as follows: 4 pg/ml (TNF-α), 13 pg/ml (TGF-β), and 1 μg/ml (CRP).

### Markers of chronic foetal hypoxia

Because scalp blood gas analysis, the most accurate means of assessing foetal hypoxia, was not feasible in the study setting, other proxies for chronic foetal hypoxia were measured: erythropoietin (Epo) [19], and cortisol and corticotrophin-releasing hormone (CRH) [20]. Maternal peripheral, placental, and cord plasma erythropoietin (Epo) levels were assayed by ELISA according to manufacturer's instructions (IBL, Hamburg, Germany). Placental and cord cortisol (CRT) and placental CRH were determined by ELISA as well (cortisol: R&D Systems, Minneapolis, MN, USA; CRH: Phoenix Pharmaceuticals, Belmont, CA, USA, respectively). Data were analysed as described above. Limits of detection were as follows: 0.4 mU/mL (EPO), 0.16 ng/ml (cortisol), and 0.19 ng/ml (CRH).

### Ethical approval

This study was approved by the College of Medicine Research Committee, University of Malawi, and the Institutional Review Boards of the University of Michigan and the University of North Carolina.

### Statistical analyses

Data were entered in MS-Access and analyses were performed using SPSS v.10 and Intercooled Stata 8.0. Skewed data were log or (log+1) transformed before certain analyses. Maternal characteristics in malaria-infected and uninfected women (Table 2) were compared by t-tests (continuous variables) and chi-squared tests (dichotomous variables). Mann-Whitney U tests were used to compare maternal and cord characteristics in malaria-infected and uninfected women (Tables 3 and 4). Bonferroni's method was used as a more conservative measure of significance for multiple comparisons [30]; p values reported reflect pre-adjustment values. The 5% significance level was used to determine significance.

**Table 1:** Malaria infections by compartment and diagnostic method

Malaria infection in any compartment		Compartment	Total cases	Placental diagnostic method (# positive cases)	Total cases
Present	42	Maternal malaria only	10		
		Maternal and placental malaria	19	Thick smear	1
				Histopathology	5
				Both	13
		Placental malaria only	13	Thick smear	0
				Histopathology	4
				Both	9
Absent	48				
Total	90				

**Table 2:** Maternal characteristics in women with and without maternal peripheral malaria infection

	Malaria-infected mean ± STD (n) * total n: 29*	Malaria-uninfected mean ± STD (n) * total n: 61*	
Age	20.9 ± 3.2 (29)	22.8 ± 5.2 (61)	t = 2.11, **p = 0.038**
Parity	1.5 ± 0.9 (29)	2.1 ± 1.5 (61)	t = 2.26, **p = 0.026**
Number of antenatal clinic visits	4.7 ± 2.0 (29)	5.6 ± 2.3 (61)	t = 1.78, *p = 0.079*
Maternal weight (kg)	56.4 ± 8.4 (29)	59.0 ± 7.4 (61)	t = 1.51, p = 0.136
Maternal height (cm)	154.6 ± 6.0 (29)	155.3 ± 4.9 (61)	t = 0.60, p = 0.552
BMI	23.6 ± 2.9 (29)	24.4 ± 2.6 (61)	t = 1.44, p = 0.153
MUAC (cm)	23.7 ± 2.9 (16)	24.6 ± 2.2 (38)	t = 1.33, p = 0.190
Took iron supplements	86% (25/29)	93% (55/59)	χ^2 ^= 1.16, p = 0.282
Took antimalarial tablets	83% (24/29)	95% (55/58)	χ^2 ^= 3.37, *p = 0.066*
Slept under mosquito net	17% (5/29)	31% (18/58)	χ^2 ^= 1.89, p = 0.169
8^th ^grade education or less	48% (14/29)	52% (32/61)	χ^2 ^= 0.14, p = 0.711
Lived in a mud-walled house	77% (20/26)	69% (35/51)	χ^2 ^= 0.58, p = 0.446
Mother self or formally employed	21% (6/29)	20% (12/61)	χ^2 ^= 0.01, p = 0.910
Father self or formally employed	92% (23/25)	86% (44/51)	χ^2 ^= 0.53, p = 0.468

**Table 3:** Mann-Whitney U test of effect of maternal peripheral malaria on maternal and neonatal haematological and inflammatory status

	**Infected median (IQR^a^) *n (total n: 29)***	**Uninfected median (IQR) *n (total n: 61)***	**p**
**Maternal**			
Hb (g/dL)	10.8 (10.1 – 11.2) *29*	12.1 (11.1 – 13.1)*61*	**<0.001^b^**
sTfR (μg/ml)	5.7 (3.8 – 9.2) *29*	6.6 (4.9 – 9.7) *61*	0.460
ferritin (ng/ml)	95.3 (42.3 – 180.8) *29*	16.5 (6.0 – 33.1) *61*	**0.001^b^**
TNF-α (pg/ml)	22.9 (7.9 – 72.1) *29*	1.8 (0.0 – 24.4) *61*	**0.001^b^**
TGF-β (ng/ml)	11.0 (6.0 – 15.9) *29*	15.5 (9.6 – 18.3) *55*	**0.027**
CRP (μg/ml)	85.0 (59.2 – 195.4) *29*	7.6 (2.8 – 26.2) *61*	**<0.001^b^**
Epo (mU/L)	60.6 (41.0 – 98.5) *29*	32.7 (21.6 – 52.6) *61*	**0.001^b^**
**Cord**			
Hb (g/dL)	15.9 (14.5 – 16.9) *22*	15.6 (14.5 – 17.1) *48*	0.849
sTfR (μg/ml)	6.7 (4.8 – 9.9) *29*	7.8 (4.9 – 9.7) *60*	0.430
ferritin (ng/ml)	135.5 (66.7 – 276.3) *29*	99.0 (39.4 – 158.7) *60*	**0.040**
TNF-α (pg/ml)	1.0 (0.0 – 8.4) *28*	2.7 (0.0 – 16.6) *59*	0.371
TGF-β (ng/ml)	17.8 (13.5 – 24.0) *15*	27.7 (20.7 – 31.9) *32*	**0.019**
CRP (μg/ml)	1.5 (0.7 – 1.7) *28*	1.0 (0.0 – 1.6) *61*	0.169
Epo (mU/L)	27.1 (13.4 – 58.9) *29*	25.8 (12.5 – 39.1) *61*	0.641

^a^IQR: Inter-quartile range

**^b ^**Significant after Bonferroni's adjustment for multiple comparisons.

## Results

### Study population

Of the 90 study participants, 29 had maternal peripheral malaria infections, 32 had placental malaria infections, and 42 had infections in one or both compartments. No cord blood malaria was identified in this study. Table 1 cross-references malaria infections by compartment and diagnostic method.

Table 2 shows the characteristics of study enrollees classified by maternal peripheral malaria. All women were HIV negative. Women with and without peripheral malaria infections were similar in terms of age and anthropometric measures. However, malaria-infected women were younger, of lower parity, and tended to have fewer antenatal clinic visits (Table 2). Socioeconomic status, as measured by educational level, house construction, and employment status, did not differ between the groups (Table 2). When women were alternatively classified by placental malaria status, there were again no significant differences in study participant characteristics (data not shown). However, when reclassified according to malaria infection in any compartment (maternal and/or placental), malaria-infected women had significantly lower BMI (23.4 ± 4.0 vs. 24.8 ± 2.4; p = 0.02) and fewer antenatal clinic visits (4.8 ± 1.8 vs. 5.7 ± 2.5; p = 0.045); no other characteristics differed between the groups (data not shown).

Data on the effects of malaria on maternal and foetal markers of haematological status, inflammation, and chronic hypoxia are presented in two tables: Table 3 presents the effects of maternal peripheral malaria on the markers, and Table 4 presents the effects of placental malaria.

**Table 4:** Mann-Whitney U test of effect of placental malaria on markers of neonatal haematological, inflammatory, and hypoxic status

	**Infected median (IQR^a^) *n (total n: 32)***	**Uninfected median (IQR) *n (total n: 58)***	
**Cord**			
Hb (g/dL)	15.4 (13.7 – 16.8) *25*	16.0 (15.0 – 17.1) *45*	p = 0.128
sTfR (μg/ml)	6.7 (5.1 – 9.9) *32*	7.7 (4.3 – 9.6) *58*	p = 0.765
ferritin (ng/ml)	129.0 (61.9 – 248.6) *32*	101.0 (41.2 – 210.8) *57*	p = 0.184
TNF-α (pg/ml)	1.7 (0.0 – 5.6) *30*	2.7 (0.0 – 15.9) *57*	p = 0.552
TGF-β (ng/ml)	19.5 (15.6 – 30.3) *16*	26.8 (17.9 – 31.8) *31*	p = 0.080
CRP (μg/ml)	1.5 (0.7 – 1.6) *31*	1.0 (0.0–1.6) *58*	p = 0.289
Epo (mU/L)	25.4 (13.4 – 58.1) *32*	27.5 (12.5 – 45.1) *58*	p = 0.940
Cortisol (μg/ml)	421.0 (283.5 – 538.5) *32*	511.0 (324.0 – 677.5) *53*	p = 0.425
**Placenta**			
Cortisol (μg/ml)	191.0 (155.5 – 251.0) *29*	172.0 (132.0 – 248.5) *53*	p = 0.357
CRH (pg/ml)	90.0 (42.5 – 126.4) *24*	72.3 (41.5 – 116.3) *43*	p = 0.421

^a^IQR: Inter-quartile range

The effect of malaria on haematological status differed markedly between women and neonates. Compared to their uninfected counterparts, women characterized by any of the three definitions of malaria infection tended to have significantly lower maternal Hb concentrations (maternal: Table 3; placental: p = 0.017; any: p < 0.001). Unlike maternal haematological status, neonatal characteristics were protected from the effects of maternal and placental malaria. Mean cord Hb levels did not differ between malaria-infected and uninfected women (Tables 3 and 4). In addition, malarial disease severity, including peripheral and placental parasite density, stage of disease, and deposition of fibrin and malaria pigment in placental monocytes, had no effect on neonatal Hb (data not shown). Neither cord nor maternal sTfR levels were affected by malaria infection in this study (Tables 3 and 4).

Markers of inflammation were also altered in malaria-infected women, who had significantly elevated CRP and TNF-α (maternal: Table 3; placental: CRP (p < 0.001), TNF-α (p = 0.022); any: CRP (p < 0.001), TNF-α (p = 0.004)) and lower TGF-β, although this result was not significant after Bonferroni's adjustment (maternal: Table 3; placental: NS; any: p = 0.010). There was no effect of maternal malaria on foetal TNF-α or CRP (Table 3). TGF-β was significantly decreased in the cord blood of neonates with peripheral malaria-infected mothers, although this result was not significant after Bonferroni's adjustment (Table 3). Among babies of malaria-infected women, neither increased maternal or placental parasite density nor markers of disease severity were associated with increases in measures of neonatal inflammation (data not shown). However, elevated maternal CRP in women with peripheral malaria infections was associated with significantly increased cord CRP (r = 0.48, p = 0.010) and decreased cord TGF-β (r = -0.60, p = 0.019), suggesting an association between maternal and foetal immune activation.

Malaria-infected women had significantly higher ferritin levels than uninfected women (maternal: Table 3; placental: p < 0.001; any: p < 0.001). Cord ferritin was significantly increased in the cord blood of neonates with peripheral malaria-infected mothers, although this result was not significant after Bonferroni's adjustment (Table 2). In addition, cord ferritin was correlated with increasing maternal parasite load in malaria-infected women (r = 0.474, p = 0.009). The notion that ferritin levels in this group reflected malaria-related inflammation is further supported by the inverse correlation between cord ferritin and cord TGF-β (r = -.593, p = 0.020). Additionally, primigravids with placental malaria infections, who tend to experience its inflammatory effects more acutely than multigravids, had significantly higher cord ferritin levels than did multigravid malaria-infected women (p = 0.021). However, despite the indications of immune activation in neonates of malaria-infected women, none of the cord immune markers were correlated with cord Hb or sTfR (data not shown).

Although women characterized by any of the three definitions of malaria infection tended to have significantly higher Epo levels than uninfected women (maternal: Table 3; placental: NS; any: p = 0.004), none of the proxies for foetal hypoxia examined in the study (cord Epo, placental and cord cortisol, and placental CRH) were increased in the neonates of malaria-infected women (Tables 3 and 4). Among malaria-infected women, increased maternal and placental parasitaemia and markers of disease severity were also not associated with increased levels of these factors (data not shown).

Neonatal haematological, immune and hypoxic markers were then examined in relation to poor birth outcomes that have been associated with maternal malaria, particularly low birthweight and preterm delivery. In this study, neither the presence nor the severity of maternal or placental malaria was associated with decreased birthweight or gestational age, perhaps due to the limited sample size (data not shown). Unsurprisingly, therefore, neither decreased maternal nor cord Hb was significantly associated with poorer birth outcomes in malaria-infected women (data not shown), although cord sTfR levels, which were not different between neonates of malaria-infected and uninfected women (Tables 3 and 4), were inversely associated with birthweight in women with maternal, but not placental, malaria (r = -.452, p = 0.014). In neonates of women with both maternal and placental malaria, elevated cord ferritin was correlated with lower birthweights (maternal: r = -0.427, p = 0.021; placental: r = -0.374, p = 0.035), and in those of women with maternal malaria, with shorter gestations as well (r = -0.432, p = 0.024). This association was not due to inflammation alone, since there was no association with cord CRP or TGF-β, nor to foetal haematopoiesis alone, since there was no association with cord Epo (data not shown). However, the data suggest that a combination of the haematological and inflammatory processes might be associated with poor birth outcomes commonly found following malaria infection during pregnancy.

## Discussion

In this study, the haematological status of neonates of mothers with malaria parasitaemia at delivery was not significantly impacted. Despite the effect of malaria on maternal Hb, which was expectedly lower, neonatal Hb levels were not decreased in the cord blood of malaria-infected compared to uninfected women. In addition, cord Hb values were not correlated to maternal Hb concentration in either group or severity of malaria, as described in the Results section. Although the sample size was relatively small, these results resemble more closely the results from Western studies, which tend to find unaffected cord values, than the studies of malaria- and non-malaria-exposed mother-neonate pairs in developing countries, as reviewed by Brabin [4]. It is unclear why, but the high cord haemoglobin levels observed in this study may relate to the high rates of antimalarial usage and iron supplementation in Malawian women (Table 2), which might provide a protective effect. The maintenance of cord Hb levels despite the presence of maternal anaemia and malaria suggests that the foetus has developed mechanisms to preferentially obtain sufficient iron and produce adequate amounts of red cells. Iron is transported unidirectionally from mother to foetus across a concentration gradient [31], and thus stores should be preferentially preserved in the foetus.

Ferritin is an iron storage protein that is an acute phase reactant in the presence of infection. While cord ferritin decreases in foetuses of anaemic mothers without malaria, it appears to increase in foetuses of mothers with increasing parasitaemia. Increased ferritin in the presence of inflammation has been suggested to reflect an evolutionary strategy by which the body removes bioavailable iron from the presence of parasites [32]. In foetuses of mothers with malaria, it may have an additional function: to help sequester iron obtained from the mother. Interestingly, in this study, elevated cord ferritin levels best predicted poor birth outcomes, including shorter gestation and lower birth weight. It is unclear why this association was found, particularly since no association was found in this study between birth outcomes and malaria infection or maternal anaemia, two well-established risk factors. This suggests that increased cord ferritin might be a result of the same foetal stresses that cause poor birth outcomes.

Many researchers have assumed that foetal hypoxia, rather than inflammation, mediates the effect of malaria and maternal anaemia on birth outcomes, particularly low birth weight. Despite the induction of foetal hypoxia by maternal anaemia [20,33] and the decrease in umbilical blood flow in pregnant women with malaria [34], it has not been rigorously demonstrated that chronic low-grade hypoxia causes poor birth outcomes, and it is unclear how severe anaemia must be to induce foetal hypoxia. Foetal hypoxia is difficult to quantify in nonwestern settings. In developed countries, blood gases are collected as soon as the foetal scalp is discernable exiting the birth canal. This technique is not feasible in hospitals in developing countries, but several biochemical measures in cord blood may offer proxies for foetal hypoxia. In particular, Epo, the hormone primarily responsible for regulation of erythropoiesis, is stimulated by both anaemia and hypoxia [35] and is a good marker of chronic foetal hypoxia [19,36] because Epo does not cross the placenta. Several studies have reported both adequate [37-39] and inadequate [40,41] Epo production for the level of anaemia in malaria-infected children and adults, and this question requires further investigation. In this study, maternal Epo was elevated in malaria-infected women (Table 3), but cord Epo levels were not affected by either maternal or placental malaria infections (Tables 3 and 4).

Allen [20] hypothesized that foetal stress hormones (cortisol and CRH) might mediate the link between maternal anaemia and poor birth outcomes, via the effect of anaemia-induced foetal hypoxia on these hormones. In this study, cord cortisol and placental cortisol and CRH were neither elevated in association with maternal malaria infection nor associated with poor birth outcomes. This may reflect the inadequacy of these factors as markers for chronic foetal hypoxia, either in general or in cord blood, or may simply be a result of the small sample size. On the other hand, hypoxia in the foetus may be so fleeting, as the foetus downregulates its growth in relation to available oxygen levels, that it does not trigger lasting detectable markers. This response is seen in sheep, where pregnant females with experimentally-induced anaemia evince a compensatory decrease in foetal growth but no actual evidence of hypoxia [42].

## Conclusion

In summary, cord haemoglobin levels were unchanged by maternal malaria and anaemia during pregnancy in this population. However, maternal parasitaemia induced a foetal inflammatory response, specifically lower TGF-β and higher ferritin levels. Elevated cord ferritin in turn was associated with lower birthweights and shorter gestations.

## Authors' contributions

ETA was responsible for conception and design of the study; IRB approval; conducting assays; analysis and interpretation of data; drafting the paper; and revising it for publication. JK and DDK performed many of the assays and analyses of these assays. VM designed the larger study and conducted much of the data analysis of that study that formed the foundation of this one. ET and VLM supervised patient enrollment and acquisition of data and aided in its submission to the IRB. MEM and SRM were central to the conception and design of the study, the analysis and interpretation of data, and the revision of the paper for publication. SRM was involved in the conception and design of the study; IRB approval; analysis and interpretation of data; drafting the paper; and revising the paper for publication.
